# Development and validation of a clinical predictive model for 1-year prognosis in coronary heart disease patients combine with acute heart failure

**DOI:** 10.3389/fcvm.2022.976844

**Published:** 2022-10-04

**Authors:** Xiyi Huang, Shaomin Yang, Xinjie Chen, Qiang Zhao, Jialing Pan, Shaofen Lai, Fusheng Ouyang, Lingda Deng, Yongxing Du, Jiacheng Chen, Qiugen Hu, Baoliang Guo, Jiemei Liu

**Affiliations:** ^1^Department of Clinical Laboratory, The Affiliated Shunde Hospital of Guangzhou Medical University, Foshan, China; ^2^Department of Radiology, The Affiliated Shunde Hospital of Guangzhou Medical University, Foshan, China; ^3^Department of Radiology, Shunde Hospital, Southern Medical University, Foshan, Guangdong, China; ^4^Department of Cardiovascular Medicine, The Affiliated Shunde Hospital of Guangzhou Medical University, Foshan, China; ^5^Department of Rehabilitation Medicine, Shunde Hospital, Southern Medical University, Foshan, Guangdong, China

**Keywords:** acute heart failure, major adverse cardiac events, prognosis, clinical predictive model, coronary heart disease

## Abstract

**Background:**

The risk factors for acute heart failure (AHF) vary, reducing the accuracy and convenience of AHF prediction. The most common causes of AHF are coronary heart disease (CHD). A short-term clinical predictive model is needed to predict the outcome of AHF, which can help guide early therapeutic intervention. This study aimed to develop a clinical predictive model for 1-year prognosis in CHD patients combined with AHF.

**Materials and methods:**

A retrospective analysis was performed on data of 692 patients CHD combined with AHF admitted between January 2020 and December 2020 at a single center. After systemic treatment, patients were discharged and followed up for 1-year for major adverse cardiovascular events (MACE). The clinical characteristics of all patients were collected. Patients were randomly divided into the training (*n* = 484) and validation cohort (*n* = 208). Step-wise regression using the Akaike information criterion was performed to select predictors associated with 1-year MACE prognosis. A clinical predictive model was constructed based on the selected predictors. The predictive performance and discriminative ability of the predictive model were determined using the area under the curve, calibration curve, and clinical usefulness.

**Results:**

On step-wise regression analysis of the training cohort, predictors for MACE of CHD patients combined with AHF were diabetes, NYHA ≥ 3, HF history, Hcy, Lp-PLA2, and NT-proBNP, which were incorporated into the predictive model. The AUC of the predictive model was 0.847 [95% confidence interval (CI): 0.811–0.882] in the training cohort and 0.839 (95% CI: 0.780–0.893) in the validation cohort. The calibration curve indicated good agreement between prediction by nomogram and actual observation. Decision curve analysis showed that the nomogram was clinically useful.

**Conclusion:**

The proposed clinical prediction model we have established is effective, which can accurately predict the occurrence of early MACE in CHD patients combined with AHF.

## Introduction

Acute heart failure (AHF) is a life-threatening condition characterized by acute dyspnea and Systemic congestion caused by abnormal cardiac structure or function, including acute decompensated heart failure (ADHF) or new onset of AHF (1). AHF is the leading cause of cardiogenic shock and cardiac arrest in patients, and the case fatality rate in hospitals is as high as 3–13%, seriously threatening their life safety, and their prognosis is extremely poor (2). The most common causes of AHF are coronary ischemic disease, cardiomyopathy, valvular disease, infective endocarditis, hypertensive heart disease, pulmonary heart disease, renal failure, and metabolic disorders. A significant proportion of coronary heart disease (CHD) patients develop AHF, and CHD is the primary cause of heart failure(HF). Despite recent advances in AHF management, such as advances in pharmacological treatment, cardiac devices, and specific heart failure programs, mortality remains high. The in-hospital fatality rate and 5-year mortality rate of AHF were 3 and 60%, respectively, and the rate of 1-year emergency department visits and emergency re-hospitalization was 50% (3). Uncontrolled AHF complicates clinical treatment and threatens patient safety (4). Thus, an effective and simple prediction model for AHF is urgently needed.

Acute heart failure is associated with a higher risk of death and re-hospitalization, and many researchers have attempted to develop different tools to predict adverse events in patients with AHF. Several researchers have developed risk score models to stratify HF patients, such as the Seattle Heart Failure Model (SHFM), the European Society of Cardiology (ESC) Model, and the American College of Cardiology/American Heart Association (ACC/AHA) Model (5–7). Although the predictive value of these models is widely recognized, some researchers report that these models do not necessarily predict the mortality of individual patients with HF (8). In addition to the high mortality rate, the high rate of re-hospitalization was due to major adverse cardiac events (MACE), which contributed to the poor prognostic outcomes of AHF (9, 10). According to the report (11), each AHF hospitalization resulted in cardiac dysfunction and a gradual decline in the patient’s clinical course, increasing the risk of re-hospitalization. Notably, its risk could be markedly increased in the short-term period after an AHF event (12–15). A significant proportion of AHF patients appear to be at an even higher risk of rapid HF progression and death following an acute event. Early, post-discharge follow-up and risk-tailored, intensive HF therapy may reduce AHF re-hospitalization and improve survival in these patients (16). At present, AHF prediction models are mostly used to predict mortality or long-term prognosis, while models for AHF after short-term are very rare. Evidence in the short-term prognosis prediction model of AHF demonstrates that early, coordinated, aggressive treatment reduces inpatient mortality (17). Recent several studies have shown that a number of risk factors are related to short-term mortality after discharge, including age, sex, ventricular function, management, and so on (18–21). Although some established algorithms are currently available, these studies have not been validated in Chinese populations. Therefore, an individualized prediction model is imperative for more accurate MACE prediction in AHF patients.

Nomogram is a new prognosis evaluation tool based on Cox proportional hazards regression model or logistic regression model that predicts individual disease risk graphically and is easily applied clinically. It mainly simplifies the prediction model by calculating a single estimated value of the probability of an event occurrence and provides a personalized prognosis assessment for individual patients to assist clinical decision-making (22). Compared with the traditional risk scoring system, a nomogram can integrate more risk factors, calculate the numerical probability of the target event, quantify the risk more accurately, and apply it more flexibly.

This study aimed to develop a clinical predictive model to predict the risk of short-term (1-year) adverse outcomes in CHD patients combined with AHF based on potential risk factors.

## Materials and methods

### Patients

A total of 692 CHD patients combined with AHF admitted to Shunde Hospital of Southern Medical University between January 2020 and December 2020 were selected for this study. Inclusion criteria: (1)Patients with CHD who were diagnosed with coronary artery stenosis > 70%; (2) Patients with AHF met the Chinese Guidelines for the Diagnosis and Treatment of Heart Failure 2018 (23); (3) Patients aged ≥ 18 years; and (4) Patients were classified as having grade II–IV cardiac function, by the New York Heart Association (NYHA) classification (24). Exclusion criteria: (1) Patients with congenital heart disease, cardiomyopathy, or valvular disease; (2) Patients complicated with malignant tumors; (3) Patients with hematological system diseases or autoimmune diseases; (4) Patients with prior history of cerebrovascular accident or mental illness; and (5) Patients with clinical data that were incomplete or lost to follow-up.

### Data collection

Based on previous studies (25, 26), we selected 40 risk factors that may predict MACE in CHD patients combined with AHF 1 year after discharge from the hospital, including age, sex, hypertension, diabetes, smoking, HF history, chronic kidney disease history, atrial fibrillation history, NYHA grades, systolic blood pressure, diastolic blood pressure, lipoprotein-associated phospholipase A2 (Lp-PLA2), homocysteine (Hcy), serum creatine kinase isoenzyme MB (CK-MB), troponin T, N-terminal pro B-type natriuretic peptide (NT-proBNP), D-dimer, total cholesterol, low-density lipoprotein cholesterol, high-density lipoprotein cholesterol, Triglycerides, fibrinogen degradation product, serum creatinine, uric acid, fasting plasma glucose, C-reactive protein, white blood cell count, neutrophil count, lymphocyte count, neutrophil to lymphocyte ratio, hemoglobin levels, platelet count, and medication status (such as diuretics, beta-blockers, Statins, Mineralocorticoid receptor antagonists, Angiotensin receptor enkephalinase inhibitors, calcium channel blockers, angiotensin-converting enzyme inhibitors, and angiotensin receptor blockers).

### Follow-up and grouping

Follow-up data were collected from our hospital’s electronic medical record system, patients’ outpatient records, and telephone conversations with patients or family members.

The 1-year adverse outcomes were defined as adverse cardiovascular events associated with stroke, Non-fatal myocardial infarction, cardiac death and re-hospitalization due to HF during the first 12 months of follow-up after discharge from the hospital.

Patients were divided into MACE and no MACE groups based on whether MACE occurred 1-year after discharge from the hospital.

### Model development and statistical analysis

All statistical calculations were computed using R software. The data were randomly divided into a training cohort (*n* = 484) and a validation cohort (*n* = 208) at about 7:3. Predictors of 1-year outcomes were analyzed using logistic regression based on the training cohort. Step-wise regression based on the Akaike information criterion was used to further select significant variables. The receiver operating characteristic curve (ROC) and its area under the curve (AUC) were used to evaluate the step-wise regression on both the training and validation cohorts. A nomogram was formulated based on the results of logistic regression. The nomogram is based on proportionally transforming the regression coefficient into a 0–100 point scale. The sum of all variables’ points could be interpreted as a probability of belonging to a class. The predictive performance of the nomogram was measured using AUC and resampling model calibration, accompanied by the Hosmer-Lemeshow test (a significant test statistic implies that the model does not calibrate perfectly). Decision curve analysis (DCA) was used to determine the clinical usefulness of the models by calculating the net benefits at different threshold probabilities in the combined training and validation datasets. The “rms” package was used for nomogram formulation and calibration. The DCA was performed by using the “rmda” package. *P*-value < 0.05 was considered to be statistically significant.

## Results

### Baseline characteristics

The mean age of the 692 patients was 67.13 ± 15.55 years, with 400 (57.8%) males and 292 (42.2%) females. Three hundred seven-nine (54.8%) patients were diagnosed with ADHF, and 313 (45.2%) patients were diagnosed with new onset of AHF. Two hundred fifty-nine (37.4%) patients were followed up with MACE, including 23 (3.3%) patients with cardiac death and 236 (34.1%) patients with re-hospitalization cause by stroke, Non-fatal myocardial infarction, or HF. Finally, 484 patients were assigned to the training cohort, which included 181 (37.4%) patients with MACE, and 208 patients were assigned to the validation cohort, which included 78 (37.5%) patients with MACE.

Clinical data from the training and validation cohorts revealed no statistically significant differences in age, sex, disease history, laboratory tests, NYHA grades, medication status, or MACE incidence (*P* > 0.05) (Table 1).

**TABLE 1:** Baseline characteristics between patients in the training and validation cohorts.

Variables	Total (*n* = 692)	Training group (*n* = 484)	Validation group (*n* = 208)	*P-*value
Age(yrs)	67.13 ± 15.55	67.54 ± 15.96	66.17 ± 14.55	0.290
Sex (n,%)				0.897
Male	400(57.8%)	279(57.6%)	121(58.2%)	
Female	292(42.2%)	205(42.4%)	87(41.8%)	
Hypertension(n,%)				0.353
YES	447(64.6%)	318(65.7%)	129(62.0%)	
NO	245(35.4%)	166(34.3%)	79(38.0%)	
Diabetes (n,%)				0.818
YES	408(59.0%)	284(58.7%)	124(55.8%)	
NO	284(41.0%)	200(41.3%)	84(41.3%)	
Smoking (n,%)				0.703
YES	345(49.9%)	239(49.4%)	106(51.0%)	
NO	347(50.1%)	245(50.6%)	102(49.0%)	
HF (n,%)				0.878
First	313(45.2%)	218(45.0%)	95(43.7%)	
Former	379(54.8%)	266(55.0%)	113(56.3%)	
CKD (n,%)				0.140
YES	428(61.8%)	308(63.6%)	120(57.7%)	
NO	264(38.2%)	176(36.4%)	88(42.3%)	
AF (n,%)				0.341
YES	506(73.1%)	359(74.2%)	147(70.7%)	
NO	186(26.9%)	125(25.8%)	61(29.3%)	
NYHA (n,%)				0.476
≥3 Level	345(49.9%)	237(49.0%)	108(51.9%)	
<3 Level	347(50.1%)	247(51.0%)	100(48.1%)	
MACE (n,%)				0.979
YES	259(37.4%)	181(37.4%)	78(37.5%)	
NO	433(62.6%)	301(62.6%)	130(62.5%)	
Medication care				
Diuretic (n,%)				0.659
YES	520(75.1%)	365(75.6%)	154(74.0%)	
NO	172(24.9%)	118(24.4%)	54(26.0%)	
Beta-blocker (n,%)				0.899
YES	194(28.0%)	135(27.9%)	59(28.4%)	
NO	498(72.0%)	349(72.1%)	149(71.6%)	
Statin (n,%)				0.343
YES	212(30.6%)	143(29.5%)	69(33.2%)	
NO	480(69.4%)	341(70.5%)	139(66.8%)	
MRA (n,%)				0.650
YES	181(26.2%)	129(26.7%)	52(25.0%)	
NO	511(73.8%)	355(73.3%)	156(75.0%)	
ARNI (n,%)				0.133
YES	215(31.1%)	142(29.3%)	73(35.1%)	
NO	477(68.9%)	342(70.7%)	135(64.9%)	
CCB (n,%)				0.162
YES	233(33.7%)	155(32.0%)	78(37.5%)	
NO	459(66.3%)	329(68.0%)	130(62.5%)	
ACEI (n,%)				0.706
YES	206(29.8%)	142(29.3%)	64(30.8%)	
NO	486(70.2%)	342(70.7%)	144(69.2%)	
ARB (n,%)				0.771
YES	168(24.3%)	116(24.0%)	52(25.0%)	
NO	524(75.7%)	368(76.0%)	156(75.0%)	
Clinical findings				
SBP (mm Hg)	143.41 ± 17.97	143.93 ± 17.85	142.20 ± 18.21	0.244
DBP (mm Hg)	82.98 ± 12.61	83.13 ± 12.81	82.62 ± 12.81	0.623
Lp-PLA2 (ng/L)	183.01 ± 33.67	184.19 ± 35.31	180.27 ± 29.37	0.132
Hcy (umol/L)	14.93 ± 6.11	14.66 ± 5.57	15.57 ± 7.20	0.106
CK-MB (ug/L)	45.74 ± 69.70	45.81 ± 73.94	45.56 ± 59.88	0.996
TnT (ug/L)	0.32 ± 1.00	0.32 ± 1.00	0.33 ± 1.01	0.776
NT-proBNP (ng/L)	1083.99 ± 1352.04	1096.50 ± 1364.31	1054.86 ± 1325.83	0.711
D-Dimer (ug/ml)	1.80 ± 2.44	1.66 ± 1.80	2.13 ± 3.49	0.063
Total cholesterol (mg/dl)	195.23 ± 42.52	194.46 ± 42.14	197.17 ± 44.46	0.430
LDL-C (mg/dl)	112.11 ± 32.86	111.34 ± 32.09	113.66 ± 34.79	0.339
HDL-C(mg/dl)	54.51 ± 14.69	54.51 ± 14.30	55.28 ± 15.46	0.696
Triglycerides(mg/dl)	138.21 ± 98.34	139.10 ± 102.78	139.10 ± 90.37	0.985
FDP(ug/L)	13.23 ± 13.48	12.94 ± 13.41	13.94 ± 13.66	0.370
Scr(umol/L)	220.29 ± 268.13	211.21 ± 253.42	241.40 ± 299.18	0.204
Uric Acid(umol/L)	467.01 ± 191.74	471.31 ± 194.77	457.01 ± 184.58	0.369
FPG(mmol/L)	8.56 ± 5.82	8.51 ± 6.09	8.67 ± 5.17	0.727
CRP(mg/L)	43.36 ± 53.10	43.85 ± 53.44	42.20 ± 52.65	0.709
WBC(103/μl)	9.27 ± 4.87	9.09 ± 4.47	9.71 ± 5.68	0.161
NEUT(103/μl)	3.68 ± 2.14	3.67 ± 2.04	3.72 ± 2.37	0.795
LYM(103/μl)	1.29 ± 1.31	1.26 ± 0.93	1.38 ± 1.93	0.307
NLR(%)	4.29 ± 3.96	4.36 ± 4.01	4.13 ± 4.87	0.479
HGB(g/L)	106.77 ± 31.12	106.41 ± 31.49	107.61 ± 30.28	0.642
PLT(103/μl)	204.74 ± 97.41	206.39 ± 89.96	200.90 ± 113.03	0.497

HF, heart failure history; CKD, chronic kidney disease history; AF, atrial fibrillation history; NYHA, New York heart association; MACE, major adverse cardiovascular events; MRA, mineralocorticoid receptor antagonists; ARNI, angiotensin receptor enkephalinase inhibitors; CCB, calcium channel blocker; ACEI, angiotensin-converting enzyme inhibitors; ARB, angiotensin receptor blocker; SBP, systolic blood pressure; DBP, diastolic blood pressure; Lp-PLA2, lipoprotein associated phospholipase A2; Hcy, homocysteine; CK-MB, creatine kinase isoenzyme-MB; TnT, troponin T; NT-proBNP, N terminal pro B type natriuretic peptide; LDL-C, low-density lipoprotein cholesterol; HDL-C, high-density lipoprotein cholesterol; FDP, fibrinogen degradation product; Scr, serum creatinine; FPG, fasting plasma glucose; CRP, C-reactive protein; WBC, white blood count; NEUT, neutrophil count; LYM, lymphocyte count; NLR, neutrophil to lymphocyte ratio; HGB, hemoglobin; PLT, platelet count.

Table 2 compares patients and clinical characteristics in the training cohort between the MACE and No MACE groups.

**TABLE 2:** Univariate analyses of variables associated with MACE in the training cohorts.

Variables	Training group (*n* = 484)	MACE (*n* = 181)	No MACE (*n* = 303)	*P-*value
Age(yrs)	67.54 ± 15.96	66.68 ± 15.67	68.05 ± 16.13	0.361
Sex(n,%)				0.799
Male	279(57.6%)	103(56.9%)	176(58.1%)	
Female	205(42.4%)	78(43.1%)	127(41.9%)	
Hypertension(n,%)				0.542
YES	318(65.7%)	122(67.4%)	196(64.7%)	
NO	166(34.3%)	59(32.6%)	107(35.3%)	
Diabetes(n,%)				<0.001
YES	284(58.7%)	130(71.8%)	154(50.8%)	
NO	200(41.3%)	51(28.2%)	149(49.2%)	
Smoking(n,%)				0.002
YES	239(49.4%)	106(58.6%)	133(43.9%)	
NO	245(50.6%)	75(41.4%)	170(56.1%)	
HF(n,%)				<0.001
First	218(45.0%)	102(56.4%)	116(38.3%)	
Former	266(55.0%)	79(43.6%)	187(61.7%)	
CKD(n,%)				0.723
YES	308(63.6%)	117(64.6%)	191(63.0%)	
NO	176(36.4%)	64(35.6%)	112(37.0%)	
AF(n,%)				0.788
YES	359(74.2%)	133(73.5%)	226(74.6%)	
NO	125(25.8%)	48(26.5%)	77(25.4%)	
NYHA(n,%)				<0.001
≥3 Level	237(49.0%)	117(64.6%)	120(39.6%)	
<3 Level	247(51.0%)	64(35.4%)	183(60.4%)	
Medication care				
Diuretic(n,%)				0.805
YES	365(75.6%)	138(76.2%)	228(75.2%)	
NO	118(24.4%)	43(23.8%)	75(24.8%)	
Beta-blocker(n,%)				0.919
YES	135(27.9%)	50(27.6%)	85(28.1%)	
NO	349(72.1%)	131(72.4%)	218(71.9%)	
Statin(n,%)				0.754
YES	143(29.5%)	55(30.4%)	88(29.0%)	
NO	341(70.5%)	126(69.6%)	215(71.0%)	
MRA(n,%)				0.312
YES	129(26.7%)	53(29.3%)	76(25.1%)	
NO	355(73.3%)	128(70.7%)	227(74.9%)	
ARNI(n,%)				0.522
YES	142(29.3%)	50(27.5%)	92(30.4%)	
NO	342(70.7%)	131(72.4%)	211(69.6%)	
CCB(n,%)				0.551
YES	155(32.0%)	55(30.4%)	100(33.0%)	
NO	329(68.0%)	126(69.6%)	203(67.0%)	
ACEI(n,%)				0.853
YES	142(29.3%)	54(29.8%)	88(29.0%)	
NO	342(70.7%)	127(70.2%)	215(71.0%)	
ARB(n,%)				0.309
YES	116(24.0%)	48(26.5%)	68(22.4%)	
NO	368(76.0%)	133(73.5%)	235(77.6%)	
Clinical findings				
SBP(mm Hg)	143.93 ± 17.85	144.37 ± 15.20	143.67 ± 19.29	0.660
DBP(mm Hg)	83.13 ± 12.81	84.04 ± 11.41	82.59 ± 13.57	0.208
Lp-PLA2(ng/L)	184.19 ± 35.31	206.34 ± 35.51	170.96 ± 27.79	<0.001
Hcy(umol/L)	14.66 ± 5.57	16.81 ± 5.59	13.38 ± 5.16	<0.001
CK-MB(ug/L)	45.81 ± 73.94	52.11 ± 105.95	42.06 ± 44.90	0.148
TnT(ug/L)	0.32 ± 1.00	0.27 ± 0.34	0.34 ± 1.23	0.477
NT-proBNP(ng/L)	1096.50 ± 1364.31	1481.60 ± 1613.94	866.46 ± 1132.45	<0.001
D-Dimer(ug/ml)	1.66 ± 1.80	1.89 ± 1.2.08	1.53 ± 1.59	0.063
Total cholesterol(mg/dl)	194.46 ± 42.14	193.30 ± 40.59	195.23 ± 42.91	0.633
LDL-C(mg/dl)	111.34 ± 32.09	110.95 ± 28.99	111.34 ± 34.02	0.844
HDL-C(mg/dl)	54.51 ± 14.30	52.96 ± 13.53	55.67 ± 15.08	0.034
Triglycerides(mg/dl)	139.10 ± 102.78	148.84 ± 118.72	132.90 ± 90.37	0.109
FDP(ug/L)	12.94 ± 13.41	11.60 ± 11.31	13.73 ± 14.91	0.065
Scr(umol/L)	211.21 ± 253.42	206.89 ± 229.67	213.79 ± 266.93	0.772
Uric Acid(umol/L)	471.31 ± 194.77	493.12 ± 172.47	458.28 ± 206.13	0.047
FPG(mmol/L)	8.51 ± 6.09	9.02 ± 8.56	8.20 ± 3.92	0.154
CRP(mg/L)	43.85 ± 53.44	38.00 ± 51.82	47.34 ± 54.02	0.060
WBC(103/μl)	9.09 ± 4.47	8.40 ± 4.11	9.50 ± 4.63	0.009
NEUT(103/μl)	3.67 ± 2.04	3.67 ± 2.09	3.67 ± 2.01	0.795
LYM(103/μl)	1.26 ± 0.93	1.29 ± 0.77	1.25 ± 1.01	0.983
NLR(%)	4.36 ± 4.01	4.08 ± 3.64	4.53 ± 4.20	0.588
HGB(g/L)	106.41 ± 31.49	109.50 ± 31.30	104.57 ± 31.52	0.095
PLT(103/μl)	206.39 ± 89.96	207.43 ± 83.69	205.77 ± 93.64	0.845

HF, heart failure history; CKD, chronic kidney disease history; AF, atrial fibrillation history; NYHA, New York heart association; MRA, mineralocorticoid receptor antagonists; ARNI, angiotensin receptor enkephalinase inhibitors; CCB, calcium channel blocker; ACEI, angiotensin-converting enzyme inhibitors; ARB, angiotensin receptor blocker; SBP, systolic blood pressure; DBP, diastolic blood pressure; Lp-PLA2, lipoprotein associated phospholipase A2; Hcy, homocysteine; CK-MB, creatine kinase isoenzyme-MB; TnT, troponin T; NT-proBNP, N terminal pro B type natriuretic peptide; LDL-C, low-density lipoprotein cholesterol; HDL-C, high-density lipoprotein cholesterol; FDP, fibrinogen degradation product; Scr, serum creatinine; FPG, fasting plasma glucose; CRP, C-reactive protein; WBC, white blood count; NEUT, neutrophil count; LYM, Lymphocyte count; NLR, neutrophil to lymphocyte ratio; HGB, hemoglobin; PLT, platelet count; P-value means difference between MACE group and No MACE group.

### Prediction model development and validation

**FIGURE 1:**
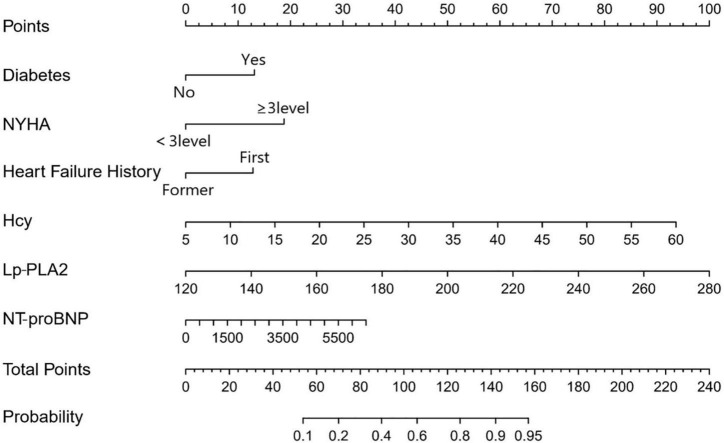
Nomogram for 1-year MACE prognosis in coronary heart disease (CHD) patients combined with AHF. The nomogram was developed in the primary cohort, with the Diabetes, HF history, NYHA ≥ 3, NT-proBNP, Lp-PLA2, and Hcy incorporated.

**FIGURE 2:**
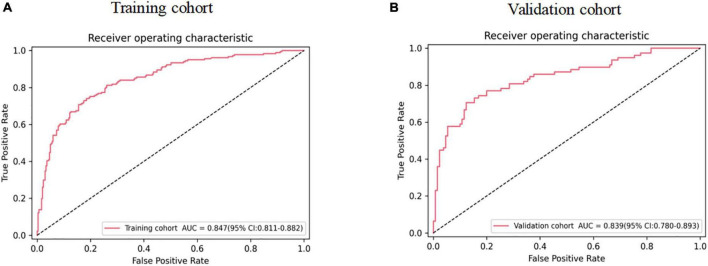
The ROC of nomogram in panels **(A,B)** training and validation cohorts. ROC, receiver operating characteristic curve.

**FIGURE 3:**
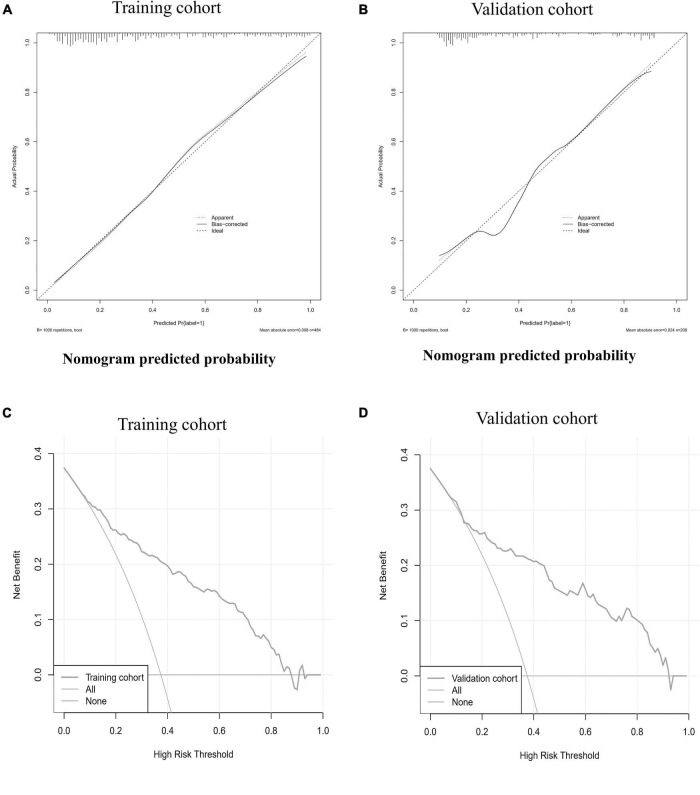
The calibration curve and decision curve of the nomogram in training and validation cohorts. The calibration curves of the nomogram in the training cohort **(A)** and validation cohort **(B)** are reported. The x-axis is the nomogram predicted probability and the y-axis is the actual probability. The prediction performance can be measured by the difference of the fitted curve and slope 1 line (diagonal 45-degree line). The diagonal dotted line represents a perfect prediction by an ideal model. The solid line represents the performance of the nomogram, of which a closer fit to the diagonal dotted line represents a better prediction. Decision curve analysis for the nomogram in the training cohort **(C)** and validation cohort **(D)** are showed. The y-axis measures the net benefit. The red line represents the nomogram. The blue line represents all patients had MACE. The orange line represents the assumption that no patients had MACE. The decision curve showed that if the threshold probability of a patient uses the nomogram offered a net benefit over the “happen-all” or “happen-none” strategy at a threshold range form 8–88 to 8–92%, respectively in the training cohort and validation cohort.

Six predictors, including diabetes, HF history, NYHA ≥ 3, NT-proBNP, Lp-PLA2, and Hcy, were selected by step-wise regression based on the Akaike information criterion (Table 3). The above independent predictors were incorporated into the nomogram (Figure 1). The nomogram for 1-year MACE prognosis prediction indicated an AUC of 0.847 [95% confidence interval (CI): 0.811–0.882], a sensitivity of 0.740, a specificity of 0.815, and an accuracy of 0.787 in the training cohort (Figure 2A and Table 4). Relatively, in the validation cohort, the nomogram showed an AUC of 0.839 (95% CI: 0.780–0.893), a sensitivity of 0.705, a specificity of 0.877, and an accuracy of 0.812 (Figure 2B and Table 4). Figures 3A,B depicts the nomogram calibration curve, demonstrating good agreement between prediction by nomogram and actual observation in the training and validation cohorts. The Hosmer-Lemeshow test produced a non-significant statistic (χ^2^ = 6.522, *P* = 0.589 in the training cohort and χ^2^ = 5.648, *P* = 0.687 in the validation cohort, respectively), indicating no perfect fit deviation.

**TABLE 3:** Multivariate analysis of independent risk factors associated with MACE.

Multivariable analysis						
Variables	B	S.E	Wals	OR	95% CI	*P*-value
Diabetes	0.627	0.25	6.32	1.873	1.148-3.055	0.012
Heart failure history	0.634	0.24	6.984	1.885	1.178-3.016	0.008
NYHA ≥ 3Level	0.990	0.239	17.107	2.692	1.684-4.303	<0.001
NT-proBNP	0.003	0.001	8.136	1.003	1.001-1.004	0.004
Lp-PLA2	0.031	0.004	60.765	1.031	1.023-1.039	<0.001
Hcy	0.088	0.024	13.246	1.092	1.042-1.145	<0.001

NT-proBNP, N terminal pro B type natriuretic peptide; Lp-PLA2, lipoprotein associated phospholipase A2; Hcy, homocysteine; OR, odds ratio; CI, confidence interval.

**TABLE 4:** Predictive value of nomogram in training group and validation group.

Model	Accuracy	Sensitivity	Specificity	F1_score	AUC (95% CI)
**MACE**					
Training group (*n* = 484)	0.787	0.740	0.815	0.722	0.847 (0.811–0.882)
Validation group (*n* = 208)	0.812	0.705	0.877	0.738	0.839 (0.780–0.893)

AUC, area under the curve; CI, confidence interval.

### Clinical usefulness of the nomogram

In the training and validation cohort DCAs, the nomogram offered a net benefit over the “happen-all” or “happen-none” strategies at a threshold of 8–88 and 8–92%, respectively (Figures 3C,D), demonstrating that our nomogram was clinically useful. For example, in a training cohort with a threshold probability of 40%, using the clinical nomogram could provide an additional net benefit of 0.2 over the “happen-all” or “happen-none” strategy.

## Discussion

We included six independent factors associated with 1–year prognosis in CHD patients combined with AHF, including diabetes, HF history, NYHA ≥ 3, NT-proBNP, Lp-PLA2, and Hcy. The developed clinical nomogram model achieved good predictive performance in the training cohort (AUC = 0.847, 95% CI: 0.811–0.882) and validation cohort (AUC = 0.839, 95% CI: 0.780–0.893). The clinical predictive model has been clinically validated.

HF is one of the most common acute and severe diseases in internal medicine, as well as the final stage of the progression of various cardiovascular disorders. It has been reported that a significant number of patients with AHF are re-hospitalized after discharge due to recurrent symptoms or die within months of being discharged from the hospital (27). Our study showed that during 1-year post-discharge follow-up, 259 patients (37.4%) were followed up with MACE, including 23 (3.3%) patients with cardiac death and 236 (34.1%) patients with re-hospitalization cause by stroke, Non-fatal myocardial infarction, or HF. This result is consistent with the findings of the European Heart Organization and the American Heart Association (28–30). They concluded that AHF represented high-risk patients with higher mortality and likelihood of re-hospitalization during the same follow-up period than chronic stable HF. The treatment goal of HF is not only to improve symptoms and quality of life but also to prevent and delay the development of cardiac remodeling by targeting the mechanism of cardiac remodeling to reduce the mortality and hospitalization rate of patients with HF (31). Delayed diagnosis of AHF worsens prognosis by increasing the time to initiate initial treatment, and this delay may be associated with increased morbidity and mortality (32). That is why the individualized prediction of AHF is critical.

According to the 2018 Guidelines for the Diagnosis and Treatment of Heart Failure in China, the associated factors for poor prognosis of patients with HF are decreased left ventricular ejection fraction, continuously increased natriuretic peptide levels, deterioration of NYHA cardiac function grading, hyponatremia, decreased hematocrit value, chronic hypotension, resting tachycardia, and renal insufficiency (33). However, studies on identifying short-term (1-year) prognosis predictors of CHD patients combined with AHF are scarce. Inspired by previous studies, our nomogram model is based on the available clinical high performance in predicting the 1-year prognosis of CHD patients combined with AHF, including diabetes, HF history, NYHA ≥ 3, NT-proBNP, Lp-PLA2, and Hcy. Diabetes can increase the mortality rate in patients with HF by 50–100% (34, 35). Mebazaa et al. have shown that hyperglycemia has a poor short-term prognosis for AHF and can exacerbate its progression. Lassus et al. suggested that both all-cause mortality and cardiovascular mortality are lower in patients with new-onset AHF than in patients with acutely decompensated chronic heart failure (ADCHF) (36, 37). Studies have shown that patients with ADCHF have a longer course of CHD and more significant cardiac remodeling. ADCHF will further aggravate the damage to myocardial cells and extracellular matrix damage, resulting in aggravated cardiac remodeling and deterioration of cardiac function (36, 38). NT-proBNP is a bioactive lysis product of brain natriuretic peptide (BNP), produced and released by ventricular myocytes under ventricular wall stress (39). Causes of BNP release during AHF include myocardial cell extension, tissue ischemia, and myocardial remodeling (40, 41). Many experiments confirmed that NT-probNP is a predictor of the prognosis of HF. And our studies showed that NT-probNP is an independent risk factor for short-term prognosis. Moreover, the higher the NT-proBNP index of AHF patients, the more severe the symptoms of HF, and the worse the short-term and long-term prognosis (42). Belkin et al. suggested that NYHA ≥ III is a risk factor for AHF (43). BNP levels and NT-proBNP concentrations of AHF patients increased gradually as their NYHA cardiac classification increased, and the differences between the III/IV and II groups were statistically significant. Sheng et al. have suggested that LP-PLA2 is an independent predictor of vascular endothelial injury, which is associated with the occurrence and prognosis of both ischemic and non-ischemic heart failure (44). High levels of Hcy are independent risk factors for HF induced by CHD (45). Studies showed that the 5- or 3-year mortality rate of HF patients with high levels of Hcy is significantly higher than that of patients with normal levels of Hcy, and Hcy levels may be an independent predictor of the long-term prognosis of patients with HF (46, 47). Our study suggests that LP-PLA2 and Hcy were independent factors for 1-year prognosis of AHF. However, the effect of Hcy and LP-PLA2 levels on the short-term prognosis of HF has not been widely reported. Thus, larger sample size studies are required to confirm the findings of this study.

As an intuitive expression of the analysis results of a statistical model, a nomogram is more concise and effective in quantifying risks. Studies have confirmed that the nomogram has a good application effect in predicting the risk of acute kidney injury in patients with acute myocardial infarction after percutaneous coronary intervention (48) and in identifying the risk of HF in patients with CHD (49). However, no studies on the development of 1-year prognosis risk in CHD patients combined with AHF have been published. In our study, 6 independent risk factors affecting the occurrence of MACE in CHD patients combined with AHF after discharge from the hospital were screened out by step-wise regression, and a personalized nomogram prediction model was constructed. The AUC values for the training and validation cohorts were 0.847 (95% CI: 0.811–0.882) and 0.839 (95% CI: 0.780–0.893), with sensitivity and specificity of 0.740 and 0.705 and 0.815 and 0.877, respectively, suggesting that the nomogram had good prediction ability. It is better than the prediction model reported by Kadoglou et al., which 1-year prediction model has an AUC value of 0.698 (25). The Hosmer-Lemeshow test confirmed that the deviation between the risk prediction value of the nomogram and the actual observed value was not statistically significant (χ^2^ = 6.522, *P* = 0.589 in the training cohort and χ^2^ = 5.648, *P* = 0.687 in the validation cohort, respectively). Moreover, the calibration curve shows the mean absolute error of the internal verification of the nomogram, which is 0.008 and 0.024, respectively, indicating that the nomogram has good calibration and pretest uniformity. DCA curve analysis showed that the nomogram had good clinical applicability. Meanwhile, the prediction indexes required for constructing the nomogram are all derived from the clinical data of patients during hospitalization, which is easy to obtain and does not require complex calculation transformation. In conclusion, the nomogram for predicting the risk of 1-year MACE in CHD patients combined with AHF after discharge from the hospital has a high predictive value and clinical application value, and targeted preventive measures can be formulated for patients to reduce the occurrence of MACE in CHD patients combined with AHF.

This study has several limitations. Firstly, this was a single-center and retrospective study with a small sample size. The training and validation cohorts’ data in this study were based on the researcher’s location. Therefore, the 1-year MACE clinical predictive model must be verified in additional regional databases. Secondly, we did not analyze the occurrence of MACE in patients in the two subgroups of newly developed AHF and ADCHF due to the smaller sample size and heterogeneity of baseline data, which would lead to decreased accuracy of results. Finally, the clinical relevance or applicability of the nomogram we constructed should be validated in a prospective cohort of patients.

## Conclusion

In conclusion, this study identified 6 predictors of 1-year prognosis of MACE in CHD patients combined with AHF. A clinical predictive model is established based on the predictors to identify who will experience short-term MACE, to enhance more effective clinical intervention for CHD patients combined with AHF. DCA confirmed the clinical usefulness of the nomogram.

## Data availability statement

The raw data supporting the conclusions of this article will be made available by the authors, without undue reservation.

## Ethics statement

Written informed consent was obtained from the individual(s) for the publication of any potentially identifiable images or data included in this article.

## Author contributions

XH, SY, JL, BG, FO, and QH: conception and design. XH, QZ, XC, JP, JC, SL, and FO: acquisition of data. XH, SY, BG, FO, LD, YD, JC, and QH: analysis and interpretation of data. XH, SY, BG, and JL: drafting or revising the article. All authors contributed to the article and approved the submitted version.
